# A Simple High-Throughput Technology for Microorganism Detection and Quantitative Analysis

**DOI:** 10.3390/foods13182954

**Published:** 2024-09-18

**Authors:** Liping Wang, Ziyun Wu

**Affiliations:** School of Agriculture and Biology, Shanghai Jiao Tong University, Shanghai 200030, China; lorraine2014@sjtu.edu.cn

**Keywords:** colorimetric, high throughput, microbial counts, trace sample, microplate reader

## Abstract

Normal and damaged microorganisms are related to food safety. The colony-forming unit (CFU) assay and viability of microorganisms have broad applications in food. Traditionally, the CFU assay has been the benchmark for assessing microbial viability across various fields. However, the normal and damaged microorganisms cannot be distinguished. Here, we introduce an improved technology for foods that uses a visible absorbance microplate reader platform for high-throughput quantitative analysis of microbial lag time, doubling time, and CFU. This platform utilizes a 96-well plate and a microplate reader to accurately determine the viable cell number from a five-microliter sample. It boasts the capability to measure a dynamic range spanning from five to seven orders of magnitude, significantly reducing the time required by over 20-fold in comparison to traditional spread plate methods. Additionally, it demonstrates a remarkable ability to detect a single cell within a well. A mild temperature treatment for cell viability detection was implemented and was able to reflect the real microbial quality. Consequently, the high-throughput method as an improved technology provides essential technical support for microbial detection.

## 1. Introduction

Measuring microbial viability has diverse applications across a broad range of fields where microbiology plays a crucial role, including the food industry, agriculture, public health, environmental science, pharmaceuticals, and healthcare [1,2]. Traditionally, the dilution and plating method has been the standard procedure for quantifying live bacteria in terms of colony-forming units (CFUs). Among these techniques, the spread plate method is widely recognized for its utility in microbial enumeration. This process involves dispersing a bacterial sample across an agar plate, followed by incubation to allow colony development, after which the colonies are counted [3]. To facilitate accurate colony counts, samples often require dilution. However, the manual enumeration of bacterial CFUs on agar plates not only demands considerable time but also subjects the process to potential inaccuracies [4].

Image analysis for CFU counts is a useful approach in microbiological processing. A new segmentation algorithm alongside specialized colony counter hardware, a phase-contrast microscope, and geometric viability assay (GVA) were developed for counting bacterial CFUs [4,5,6]. The methodologies outlined for bacterial CFU enumeration significantly streamline labor, time, and resource expenditure to varying extents. Despite the adaptability of imaging methods to high-throughput detection, distinguishing living from dead cells necessitates staining [7]. The microbial biosensors, including an ultra-sensitive surface-enhanced Raman scattering biosensor utilizing gold nanostars [8], a colorimetric pattern and sensor array composed of metal complexes (C1–C11) based on a single azophenol dye-based receptor [9], and phosphorescent oxygen sensors [10] have gained recognition for their exceptional sensitivity and specificity [11]. The mentioned microbial sensors demonstrate a notable level of specificity in their applications.

In this study, we introduced a high-throughput microbial detection method utilizing quantitative colorimetric technology, centered around the viability assay [12,13]. Three typical strains of BY4742, OP50, and LGG were used in the experiments. Baker’s yeast (*Saccharomyces cerevisiae*) has been referred to as the *Escherichia coli* of the eukaryotic world [14]. *Escherichia coli* of OP50 and *Lactobacillus rhamnosus* are both procaryotic organisms [15]. Meanwhile, *Escherichia coli* is a Gram-negative bacterium, and *Lactobacillus rhamnosus* is a Gram-positive bacterium. They have a wide application in studies relevant to human aging and age-related diseases [16], as food for laboratory maintenance of *C. elegans*, and in the manufacturing of fermented dairy products and other fermented foods [17].

## 2. Materials and Methods

### 2.1. Materials

The wild-type yeast strain *Saccharomyces cerevisiae* BY4742 (MATα *his3*Δ1 *leu2*Δ0 *lys2*Δ0 *ura3*Δ0) was obtained from Horizon Discovery (Cambridge, UK). The *Escherichia coli* strain of OP50 was obtained from the Caenorhabditis Genetics Center (Minneapolis, MN, USA). The *Lactobacillus rhamnosus* strain of LGG was a gift from Professor Xianming Shi at Shanghai Jiao Tong University. The culture of each strain was kept in storage at −80 °C. The yeast extract peptone dextrose (YPD) medium was from Solarbio life science (Beijing, China), LB Lennox broth was from Sangon biotech (Shanghai, China), and MRS broth was from Qingdao Hope Bio-Technology Co., Ltd. (Qingdao, China).

### 2.2. Optimization of Medium for Three Strains

The cells of BY4742, OP50, and LGG were prepared by streaking a strain from frozen stocks onto YPD, LB, and MRS agar plates, respectively. After incubating the cells until colonies appeared, a single colony was picked and inoculated into a 1.0 mL liquid medium in a 5 mL tube and cultured at 30 °C for 2 days in a flat incubator with shaking at 200 rpm. The 2-day cultures were diluted with the corresponding medium. The 5 μL dilutions were pipetted into each well of 96-well microplate. A total of 100 μL of medium was then added to each well. The growth curves were first analyzed in individual media, namely, YPD, LB, and MRS. Subsequently, binary mixtures were explored, comprising 90% YPD + 10% LB, 70% YPD + 30% LB, 50% YPD + 50% LB, 90% YPD + 10% MRS, 70% YPD + 30% MRS, and 50% YPD + 50% MRS. Finally, ternary combinations were examined, including 80% YPD + 10% LB + 10% MRS, 60% YPD + 20% LB + 20% MRS, and 40% YPD + 30% LB + 30% MRS. The monitoring time was 20 h, and each set of experiments was repeated at least 6 times.

### 2.3. Reproducibility, Precision, and Accuracy of the High-Throughput Platform

**Figure 1:**
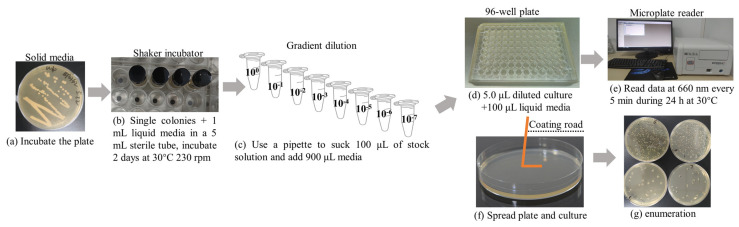
A roadmap of the platform-based colorimetric method to detect microbial counts using the high-throughput method. (**a**) Strains were activated in solid plates; (**b**) the strains were cultured in the corresponding liquid media; (**c**) the bacterial solution was gradient diluted using liquid media; (**d**) 5.0 μL of diluted culture was injected into a 96-well plate, followed the addition of 100 μL of combined media; (**e**) a commonly used microplate reader with shaking and temperature was used to read data at 660 nm every 5 min for 24 h at 30 °C; (**f**) the culturable cells were counted using the spread plate method; (**g**) enumeration.

The 2-day cultures of the three strains were diluted with medium (10^0^, 10^−1^, 10^−2^, 10^−3^, 10^−4^, 10^−5^, 10^−6^, 10^−7^). The 5 μL dilutions were pipetted into each well of a 96-well microplate. A total of 100 μL of optimized combined media was then added to each well. Each set of experiments was repeated at least 6 times. The traditional method was employed for CFU enumeration by spread plating [18]. According to the type of bacteria, an agar medium was selected and prepared. A ten-fold dilution series was diluted with the corresponding medium. The inoculum volume was 0.1 mL in terms of spreading. After the inoculum had been absorbed into the agar for 5–10 min, the plates were inverted and incubated to the desired condition. When enumerating CFUs, plates with between 20 and 300 CFUs can be used to calculate the number of CFUs/mL in the original sample. The detailed procedures are shown in Figure 1.

### 2.4. Trace Cell Detection

The 2-day cultures of OP50 were counted using the spread plate method. Subsequently, the cultures were diluted using LB medium to yield 1 and 2 cells per 5 μL. The 5 μL dilution was pipetted into each well of the 96-well microplate. A total of 100 μL of optimized medium was then added to each well. The 5 μL dilution was carefully pipetted onto an agar plate that had been divided into grids. Each set of experiments was repeated at least 12 times.

The 2-day cultures of BY4742 were counted using the spread plate method. Subsequently, the BY4742 cultures were diluted using YPD medium to yield 100, 200, 300, 400, 500, 600, and 1000 cells per 5 μL. The 5 μL of each dilution were pipetted into a 96-well microplate. A total of 100 μL of optimized medium was then added to each well. Each set of experiments was repeated at least 12 times.

### 2.5. Cell Viability Detection

The OP50 cultures were diluted to 1 cell per 5 μL with LB medium. A total of 1mL of the dilutions was added to Eppendorf tubes and heated in a metal bath. The heating temperatures were 50 °C and 60 °C, and the heating times were 15, 30, and 45 min. When the heating was finished, the dilutions of 5 μL were pipetted into a 96-well microplate. A total of 100 μL of optimized medium was then added to each well. The 5 μL dilutions were carefully pipetted onto an agar plate that had been divided into grids. Each set of experiments was repeated at least 24 times.

### 2.6. Detection Procedure of the Microplate Reader

The microorganisms were cultured using an Epoch2 microplate spectrophotometer (BioTek, Winooski, VT, USA) with a constant agitation frequency of 567 cpm at 30 °C, and the intensity of the cultured cells was monitored by recording the optical density (OD) value every 5 min over a 24 h period at 660 nm.

### 2.7. Data Analysis

The raw data were exported to Microsoft Excel by GEN5, and the OD curve was plotted. The biomass, doubling time, lag time, and viability can be calculated according to our method described previously [12,13]. It is important to note that the OD value is 0.3 for both BY4742 and OP50, and it is 0.15 for the LGG strain.

## 3. Results

### 3.1. Detection Principle of High-Throughput Method for Microbial Detection

**Figure 2:**
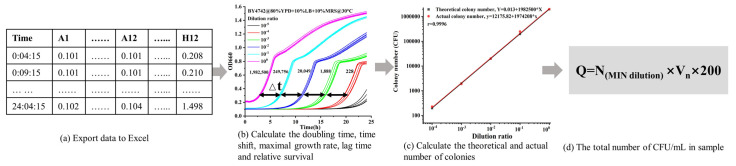
A roadmap of the platform-based colorimetric method to quantify microbial counts using the high-throughput method. (**a**) The data were exported to excel; (**b**) the formula for doubling time, lag time, and survival was calculated; (**c**) the diluted ratio and actual number of colonies were evaluated; (**d**) survival was used to determine the total CFU/mL in each sample.

The combined medium (100 μL) was inoculated into a 96-well plate containing a 5 μL sample, and a microplate reader was applied to monitor the growth curve. Specifically, the microplate reader can incubate a maximum of 96 wells/samples per assay at 30 °C and a constant agitation frequency of 567 cpm. The OD value of each well at 660 nm was recorded every 5 min for a duration of 24 h. The quantification method is shown in Figure 2. Sample serial dilution was used to establish the upper and lower detection limits. Based on the CFUs on the agar plate, the dilution ratio of CFUs can be computed with great precision. The biomass, lag time, and viability can be calculated according to our method described previously [12,13]. The minimal dilution in the linear range is defined as 100% viability. Relative survival can be obtained by measuring its viability. The CFU/mL in sample (Q) can be calculated as follows:

$$Q=N_{min\;dilution}\times V_{n}\times 200$$

where $N_{min\;dilution}$ is the CFU in the minimal dilution in the linear range, Q is the total number of CFU/mL in a sample, and $V_{n}$ is the relative survival. 

### 3.2. Effect of Different Media on the Growth of Microorganisms

**Figure 3:**
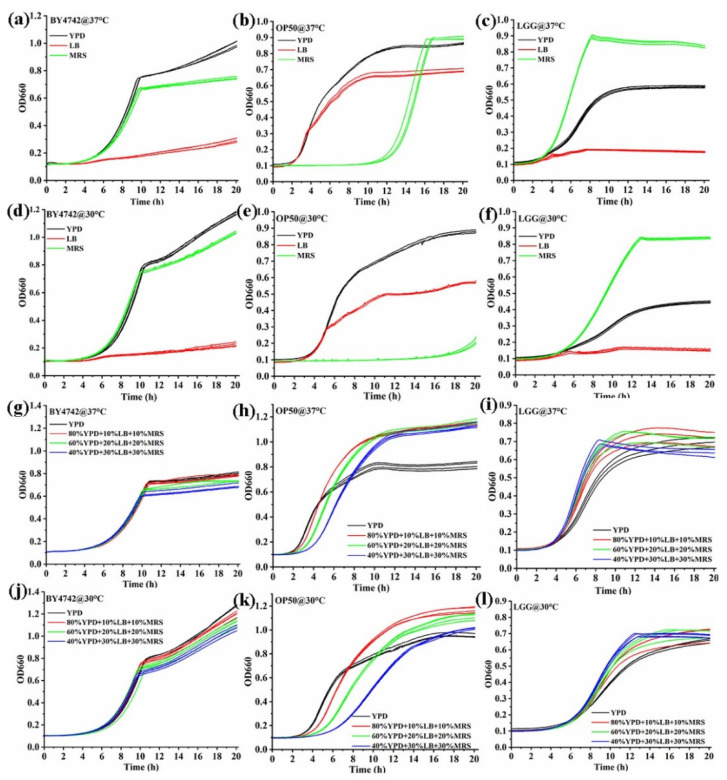
The YPD, LB, and MRS media significantly influenced the growth curves of BY4742, OP50, and LGG. (**a**) BY4742 grew better in YPD and MRS than LB at 37 °C; (**b**) OP50 grew better in YPD and LB than MRS at 37 °C; (**c**) LGG grew better in MRS and YPD than LB at 37 °C; (**d**) BY4742 grew better in YPD and MRS than LB at 30 °C; (**e**) OP50 grew better in YPD and LB than MRS at 30 °C; (**f**) LGG grew better in MRS and YPD than LB at 30 °C; (**g**) the growth curves of BY4742 at 37 °C in three combined media; (**h**) the growth curves of OP50 at 37 °C in three combined media; (**i**) the growth curves of LGG at 37 °C in three combined media; (**j**) the growth curves of BY4742 at 30 °C in three combined media; (**k**) the growth curves of OP50 at 30 °C in three combined media; (**l**) the growth curves of LGG at 30 °C in three combined media.

The majority of studies published have been concerned with the relationship between the growth of an organism and the amount of some essential nutrient in the medium [19]. Every strain typically has a preferred medium. The development of a combined medium is a key area of research for this approach because a broadly applicable medium can detect a variety of strains. The results of the study, which examined the effects of three media (YPD, LB, and MRS) and two temperatures (37 °C and 30 °C), are displayed in Figure 3a–f. The growth curves of BY4742, OP50, and LGG were significantly influenced by the effects of YPD, LB, and MRS media. In YPD medium, BY4742 and OP50 grew more effectively, and LGG could also grow. The strains’ growth tendency in all three media remained unchanged by the incubation temperature. The results indicated that YPD medium might be used in more microorganisms. Subsequently, the different proportions of two media were studied (Supporting Figure S1), with YPD accounting for 50%, 70%, and 90%, respectively. The growth curves of BY4742 and LGG could be affected when the proportion of LB was set at 50%.

Furthermore, as shown in Figure 3g–l, the different proportions of the three media were studied. Among them, the percentages of YPD were 40%, 60%, and 80%, and the percentages of LB and MRS were 30%, 20%, and 10%. Considering the growth of three strains, these results indicated that the most widely applied combination among strains of BY4742, OP50, and LGG was 80% YPD + 10% LB + 10% MRS. The doubling time and lag time were further calculated, as shown in Supporting Figure S2. For BY4742, the optimal medium (80% YPD + 10% LB + 10% MRS) had little effect on the doubling time and lag time. Even though the doubling time for OP50 was noticeably longer, the additional time was roughly 5 min. For LGG, the doubling time and lag time had no significant change with only a slight reduction.

### 3.3. Evaluation of Reproducibility, Precision, and Accuracy of the High-Throughput Method

**Figure 4:**
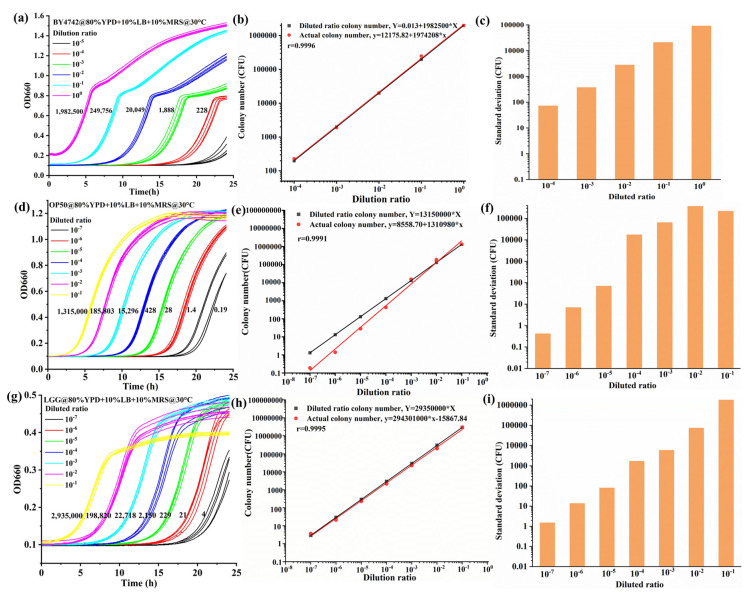
The growth curves of the three strains varied in differently diluted ratios. (**a**) The growth curves of BY4742 were different according to dilutions of 100 and 10-5; (**b**) the r value of correlation between the actual and diluted ratio CFU for different dilutions was 0.9996 in BY4742; (**c**) the standard deviation of the actual CFU for different dilutions of BY4742; (**d**) the growth curves of OP50 were different according to dilutions from 10^−1^ to 10^−7^; (**e**) The r value of correlation between the actual and theoretical CFU for different dilutions was 0.9991 in OP50; (**f**) the standard deviation of the actual CFU for different dilutions of OP50; (**g**) the growth curves of LGG were different according to dilutions from 10^−1^ to 10^−7^; (**h**) the r value of correlation between the actual and theoretical CFU for different dilutions was 0.9995 in LGG; (**i**) the standard deviation of the actual CFU for different dilutions of LGG.

To validate the platform-based colorimetric method to quantify microbial counts using a high-throughput method, the growth curves of different dilutions (10^0^–10^−7^) were investigated. For three strains (BY4742, OP50, and LGG), a 5 μL culture was inoculated into a 96-well plate, and each well was supplemented with 100 μL of optimal medium (80% YPD + 10% LB + 10% MRS). The cell growth kinetics were monitored by recording the OD value every 5 min for 24 h at 660 nm. Based on the growth curves, the viability of cells was obtained. The relative survivals were computed according to the viability. In a linear range between the dilution ratio and relative survival, the cell viability of the minimal dilution is defined as 100%, and the CFU of the minimal dilution was recorded using the spread plate. The actual CFU was calculated by the survival, and the diluted ratio CFU was calculated by the dilution times. The actual CFUs are shown in Figure 4a,d,g. The actual and diluted ratio CFUs were fitted linearly, as shown in Figure 4b,e,h. The correlations between the actual and theoretical CFUs were quite high in the three different strains: BY4742 (r = 0.9996), OP50 (r = 0.9991), and LGG (r = 0.9995). The accuracy of the high-throughput method was evaluated using the standard deviation, as shown in Figure 4c,f,i. According to the difference in strains’ doubling times, the orders of magnitude that could be detected by the high-throughput method were five, seven, and seven for BY4742, OP50, and LGG, respectively. The CFU/mL could be calculated as follows: $Q=N_{MIN\;dilution}\times V_{n}\times 200$, and the $N_{MIN\;dilution}$ values were as follows: BY4742 (N_0_ = 1,982,500), OP50 (N_−1_ = 1,315,000), and LGG (N_−1_ = 2,935,000). The platform-based colorimetric method provides high precision detection for quantifying microbial counts. In the meantime, the CFU measurements of our protocol ranged from five to seven orders of magnitude.

### 3.4. The High-Throughput Method for Microorganism Detection at Trace Cell Level

**Figure 5:**
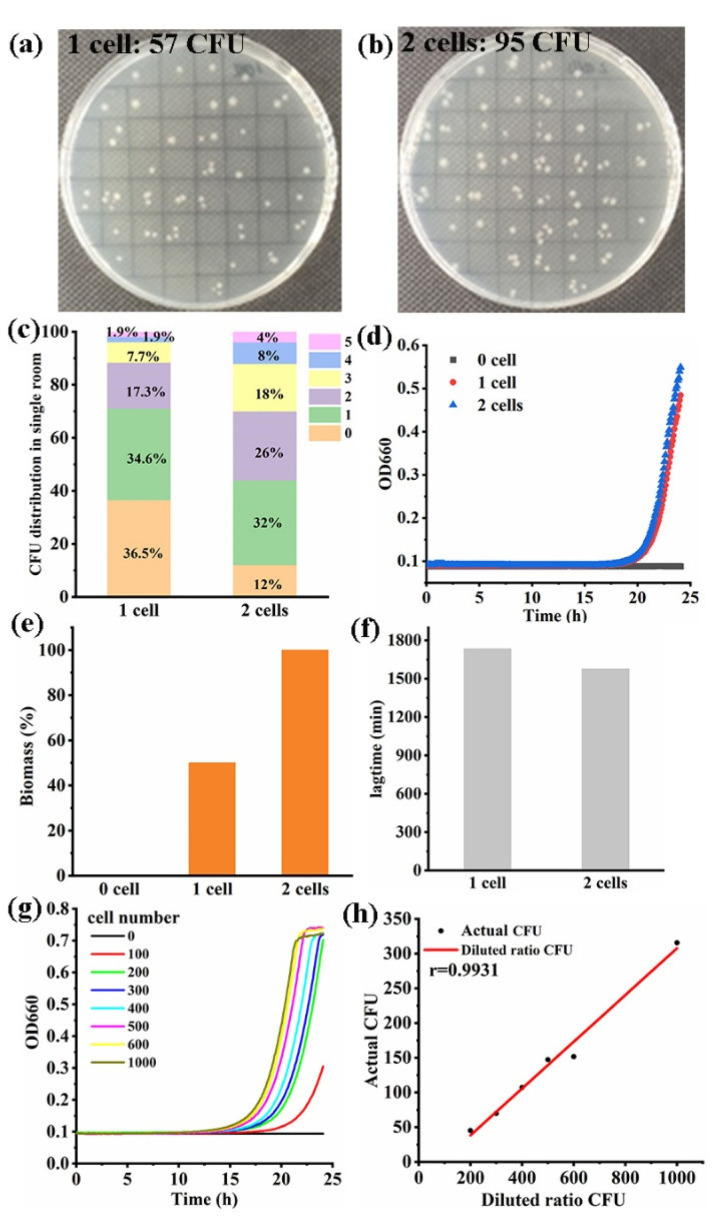
The high-throughput platform realized a trace sample for cell number detection. (**a**) The dilution of 1 cell per 5 μL was dropped onto the solid plate; (**b**) the dilution of 2 cells per 5 μL was dropped onto the solid plate; (**c**) the cell number distribution in the solid plates was counted; (**d**) the representative growth curves of single cell detection based on OP50; (**e**) the biomass result of a single cell was detected based on OP50; (**f**) the lag time of a single cell was detected based on OP50; (**g**) the representative growth curves of the trace cell were detected based on BY4742; (**h**) the correlation between the actual CFU and the diluted ratio CFU in the BY4742 sample was calculated.

Trace samples of OP50 and BY4742 were detected by the high-throughput platform. The representative results of single cell detection based on OP50, including growth curves, biomass, and lag time, are shown in Figure 5d–f. The OD value of two cells was greater than that of one cell in 24 h. The biomass of the two cells group was twice that of the one cell group. The two cells group had a shorter lag time than that of the one cell group. In order to further observe the actual cell number of OP50, the dilutions of 5 μL were dropped onto the solid plates, and the results are shown in Figure 5a, b. When the dilution of one cell per 5 μL was dropped onto the solid plate, 57 CFUs were observed in a 52-well solid plate. A total of 57 CFUs were recorded on a solid plate with 52 grids. Among all the wells, one cell per 5 μL was acquired at 34.6% probability (Figure 5c). Using the same method, the dilution of two cells per 5 μL were dropped one by one. There were 95 CFUs observed in a 50-well solid plate, and two cells per 5 μL were acquired at 26.0% probability. The representative growth curves of BY4742 (cell numbers: 0, 100, 200, 300, 400, 500, 600, and 1000) are shown in Figure 5g, and the OD value can reach 0.3 with 100 cells. The correlation between the actual CFU and the diluted ratio CFU in the trace sample was calculated, and the correlation coefficient of r was 0.9931(Figure 5h).

### 3.5. The High-Throughput Method for Cell Viability Detection

**Figure 6:**
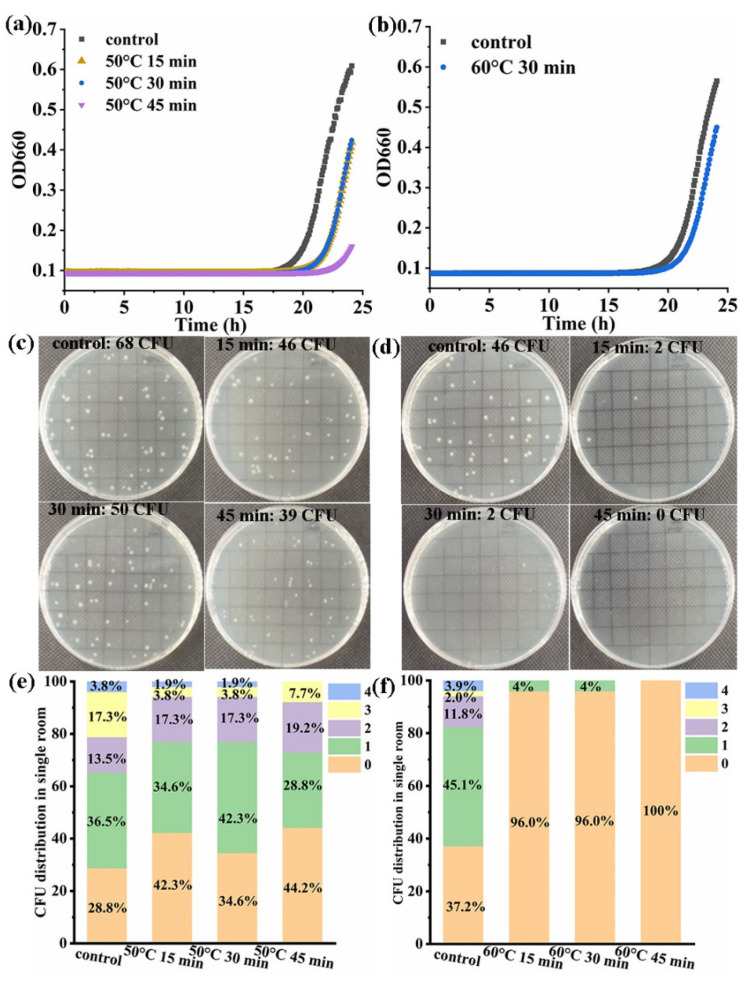
The high-throughput method achieved damaged cell detection. (**a**) The representative growth curve of OP50 was detected after 50 °C for 15 min, 30 min, and 45 min treatment; (**b**) the representative growth curve of OP50 was detected after 60 °C for 15 min, 30 min, and 45 min; (**c**) the dilution of 1 cell per 5 μL treated at 50 °C was dropped onto the solid plate; (**d**) the dilution of 1 cell per 5 μL treated at 60 °C was dropped onto the solid plate; (**e**) the cell number distribution on the solid plate was counted after 50 °C for 15 min, 30 min, and 45 min; (**f**) the cell number distribution was counted in solid medium after 60 °C for 15 min, 30 min, and 45 min.

The thermal damage degree of OP50 was further studied. The OP50 was diluted to one cell per 5 μL, and the dilutions were heated for 15 min, 30 min, and 45 min at 50 °C in a metal bath, respectively. The actual cell number of OP50 was dropped onto the solid plate, and the result is shown in Figure 6c, and the CFU number slightly decreased after heat treatment at 50 °C at different times. The CFU distributions were not significantly different on the solid plate, as shown in Figure 6e. The growth curves are shown in Figure 6a, and the results indicate that the growth curve changed with different degrees of thermal damage. But the spread plate method can only observe whether the cells are growing or not. The OP50 dilutions were heated for 15 min, 30 min, and 45 min at 60 °C in a metal bath, respectively. The actual cell number of OP50 was dropped onto the solid plate, and the CFU number changed significantly after heat treatment at 60 °C at different times, as shown in Figure 6d. The growth curves are shown in Figure 6b. The colonies did not grow on the solid plate when the cells were treated at 60 °C for 45 min. Although colonies grew on the solid plate at 60 °C for 15 min or 30 min, whether the cells were damaged or not could not be observed on the solid plate. Through the growth curve, the high-throughput method achieved damaged cell detection.

## 4. Discussion

The main advantage of the high-throughput method is a more then 20-fold reduction in time, reagent use and plastic cost as compared with the spread plate method (Table 1). To measure the time saved by the high-throughput method, we compared two main steps including the preparation time and the experimental time. More time was saved in the plating and counting step. The spread plate method required 26 h to manually plate and count 288 conditions. The high-throughput method required 1.25 h to add samples into a 96-well plate and calculate the cells. In terms of preparation, the high-throughput method was also quicker than the spread plate method. Using a microplate reader, one researcher required 1.25 h to measure the CFUs of 96 samples. The resource savings and plastic waste reduction of the two methods were then compared. Seven pipette tips per sample were required as standard in the spread plate method as each sample must be diluted and then separately transferred to a solid plate. In the high-throughput method, two pipette tips were used per sample. Compared with the spread plate method, the plastic required was reduced from 288 culture dishes to one 96-well plate in the high-throughput method and the volume of medium required was reduced from 5760 mL to 9.6 mL. Calculating the cost of pipette tips, medium, and culture plates for 96 samples, we discovered that the spread plate method was more expensive in terms of consumables, costing an average of US$0.9 per sample compared with the high-throughput method which cost US$0.08 per sample. The most time- and resource-intensive step of the classic spread plate method is the dilution series [20], and many of the plastic items are used in this process. One of the objectives of the United Nations’ sustainable development goals is responsible consumption and production [21]. The plastic items used in a microbiology laboratory with the platform-based colorimetric method to quantify microbial counts could be reduced by using high-throughput method.

The high-throughput method was established for the detection of BY4742, OP50, and LGG. A combined medium (80%YPD + 10%LB + 10%MRS) was utilized for culturing which can be easily applied to these three microorganisms. The high-throughput microbial detection assay offered significant advancements by (1) measuring viability across five–seven orders of magnitude, (2) minimizing the use of consumables, and (3) substantially reducing both the experimental duration and the cost per sample by over 20-fold and 11.25-fold, respectively, in comparison to the traditional spread plate method. According to Codex [22], microbiological risk assessment should explicitly consider the dynamics of microbiological growth, survival, and death in foods [23]. The most common imaging methods used to distinguish between dead and living cells generally include cell staining, segmentation and microscopy, which is costly and technically challenging [24]. However, the high-throughput method not only has the capability to distinguish between living and dead cells, but also to assess whether the cell is damaged or not. Flow cytometry can also determine the absolute bacterial number via staining, and the range of detection is greater than 5000 [7]. Compared to flow cytometry, the high-throughput method is capable of detecting cell numbers as low as one cell. A multiplex RT-qPCR can significantly reduce the time of analysis of culture-based methods and can specifically detect live *L. monocytogenes* [25]. In contrast to RT-PCR, the high-throughput method lacks specificity and has a wide range of applications. The platform-based colorimetric method to quantify microbial counts can be easily applied to different microbial detection in a microbiology laboratory without technical barriers. The colorimetric method used on the platform quantifies microbial counts and provides measurements of the biomass, doubling time, lag time, and survival detections. Furthermore, a trace sample can be realized by this method. The doubling time of the strains significantly influenced the detection limitation, as observed from the growth curves. Although the high-throughput method allowed for trace cell detection, it is important to note that strains with a long doubling time are not suitable for trace cell detection. Additionally, any precipitation, colored media, or turbid samples produced by bacteria in a 96-well plate are not suitable for detection.

## 5. Conclusions

In total, the high-throughput method is a reproducible, accurate, and simple way to estimate the number of living bacteria cells. It is not laborious and can be performed without any specialized training or equipment beyond a basic microplate reader. Our results showed that the high-throughput method substantially reduced operator time and reagent and plastics costs. Furthermore, the high-throughput method obtained measurements of biomass, lag time, doubling time, and viability from the growth curves. Subsequently, the number of living cells can be calculated based on the viability and survival rates. The CFU number of bacteria was easy to obtain from the survival rates and one-time spread plate count. Finally, not only can living cells be detected, but also the state of the cells can be demonstrated at a single-cell level.

## Figures and Tables

**Table 1:** Comparison of spread plate method and high-throughput method.

	Spread Plate Method	High-Throughput Method
	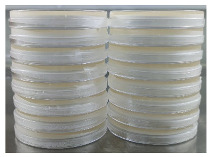	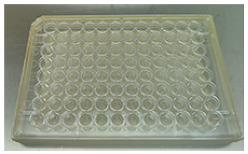
dish	288 culture dishes	one 96-well plate
volume of medium	5760 mL	9.6 mL
preparation time	5 h	2 h
incubation	48 h	24 h
count method	Manual count	Plate reader
experimental time	26 h	1.25 h
US$ per sample	0.9	0.08
result	CFU	lag time, doubling time, survival, CFU

Note: The preparation time of the spread plate method includes the medium preparation and time taken to pour, and the preparation time of high-throughput method includes medium preparation; the experimental time of the spread plate method includes dilution, spread, and counting, and the experimental time of high-throughput method includes sample addition to a 96-well plate and calculation.

## Data Availability

The original contributions presented in the study are included in the article/Supplementary Materials, further inquiries can be directed to the corresponding authors.

## Supplementary Materials

The following supporting information can be downloaded at: https://www.mdpi.com/article/10.3390/foods13182954/s1, Figure S1: Two combined media had different effects on the growth curves of BY4742, OP50, and LGG. (a) The growth curves of BY4742 at 37 °C; (b) The growth curves of BY4742 at 30 °C; (c) The growth curves of OP50 at 37 °C; (d) The growth curves of OP50 at 30 °C; (e) The growth curves of LGG at 37 °C; (f) The growth curves of LGG at 30 °C. Figure S2: The doubling time and lag time of the strains (BY4742, OP50, and LGG) were analyzed in both combined media and YPD medium. (a) The doubling time of BY4742; (b) The lag time of BY4742; (c) The doubling time of OP50; (d) The lag time of OP50; (e) The doubling time of LGG; (f) The lag time of LGG.
