# Supplementary material for: A Simple High-Throughput Technology for Microorganism Detection and Quantitative Analysis
Source: Foods. 2024 Sep 18;13(18):2954. doi: 10.3390/foods13182954 (PMC11431717; doi:10.3390/foods13182954)
Supplement: Supplementary file 1 [file foods-13-02954-s001.zip › foods-3131767-supplementary.pdf]

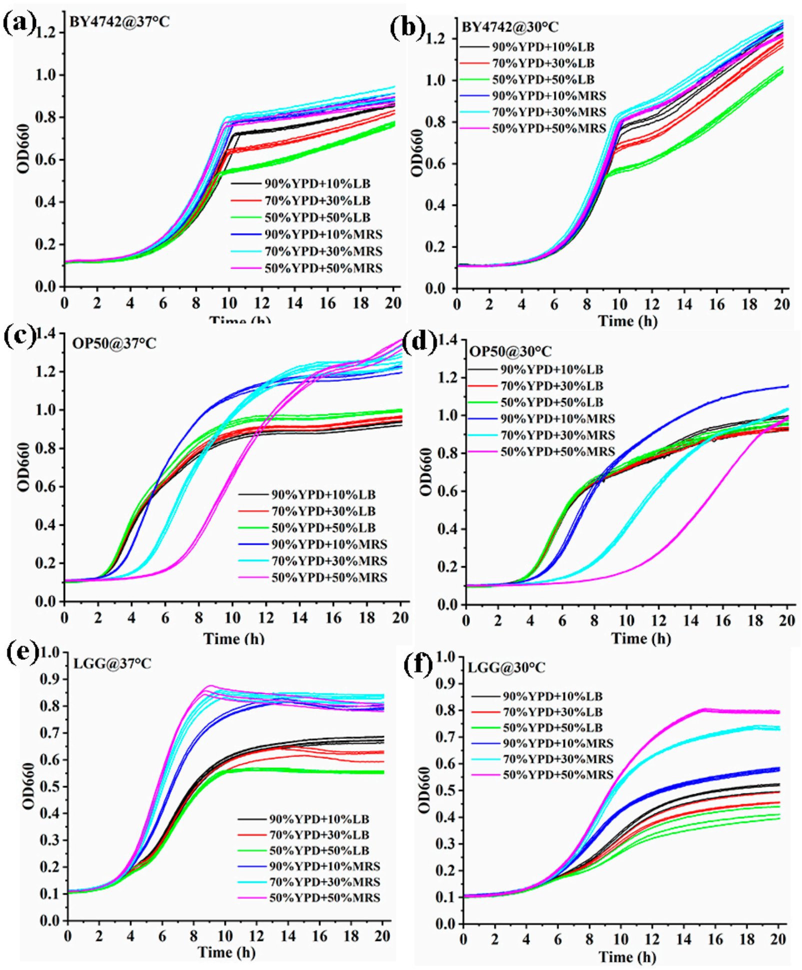

Supporting Figure S1. Two combined mediums had different effects on the growth curves of BY4742, OP50, and LGG. (a) The growth curves of BY4742 at 37°C; (b) The growth curves of BY4742 at 30°C; (c)The growth curves of OP50 at 37°C; (d) The growth curves of OP50 at 30°C; (e)The growth curves of LGG at 37°C; (f) The growth curves of LGG at 30°C.

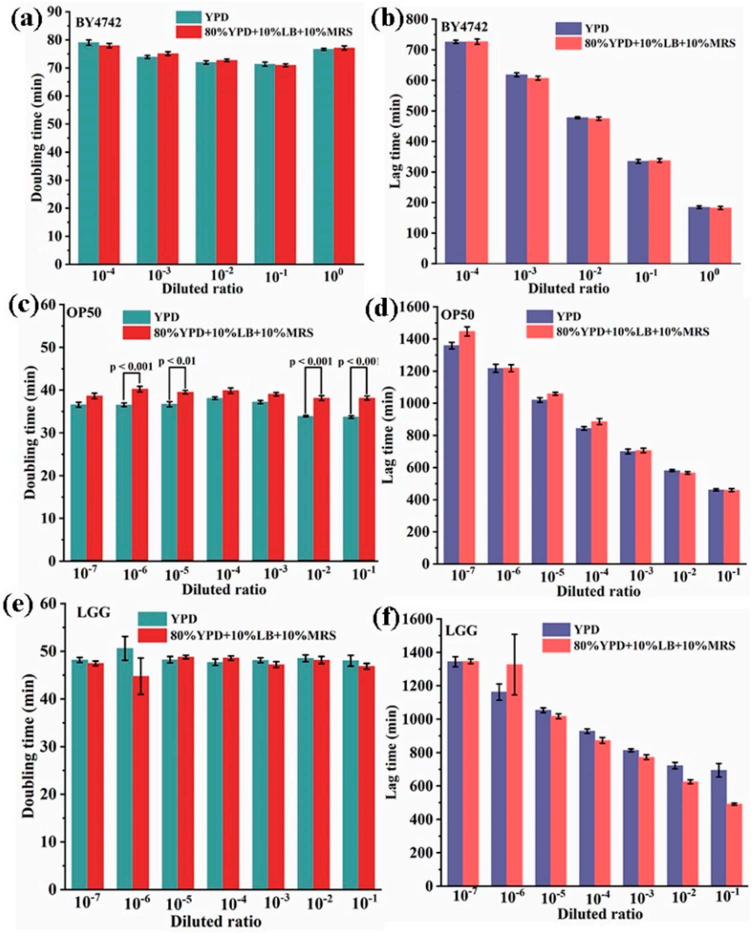

Supporting Figure S2. The doubling time and lag time of the strains (BY4742, OP50, and LGG) were analyzed in both combined mediums and YPD medium. (a) The doubling time of BY4742; (b) The lag time of BY4742; (c) The doubling time of OP50; (d) The lag time of OP50; (e) The doubling time of LGG; (d) The lag time of LGG.
